# Prognostic significance of osteopontin expression in early-stage non-small-cell lung cancer

**DOI:** 10.1038/sj.bjc.6602715

**Published:** 2005-08-09

**Authors:** L Boldrini, V Donati, M Dell'Omodarme, M C Prati, P Faviana, T Camacci, M Lucchi, A Mussi, M Santoro, F Basolo, G Fontanini

**Affiliations:** 1 Division of Surgical Pathology, Department of Surgery, University of Pisa, Pisa 56100, Italy; 2 Scuola Normale Superiore, INFN, Section of Pisa, Pisa 56100, Italy; 3 Department of Cardio-Thoracic Surgery, University of Pisa, Pisa 56100, Italy; 4 Dipartimento di Biologia e Patologia Cellulare e Molecolare, University ‘Federico II’, Naples 80131, Italy; 5 Department of Oncology, Transplants and New Technologies in Medicine, University of Pisa, Pisa 56100, Italy

**Keywords:** osteopontin (OPN), non-small-cell lung cancer (NSCLC), immunohistochemistry (IHC), disease-free survival, overall survival

## Abstract

Osteopontin (OPN) is a multifunctional protein, which has recently been shown to be linked to tumorigenesis, progression and metastasis in different malignancies. Since non-small-cell lung cancer (NSCLC)'s prognosis remains bad, with few predictors of outcome, the purpose of this study was to evaluate if OPN might be involved in NSCLC's biology and therefore represent a prognostic marker and a target for new therapeutic trials. Immunohistochemistry was used to detect OPN expression, evaluated as percentage of neoplastic cells with cytoplasmic immunoreactivity, in a wide cohort of patients with stage I NSCLC (136 cases). The median value of this series (20% of positive cells) was used as the cutoff value to distinguish tumours with low (<20%) from tumours with high (⩾20%) OPN expression. A statistically significant correlation between high levels of OPN and shorter overall (*P*=0.034) and disease-free *(P*=0.011) survival in our patients was shown. Our results support the hypothesis that high OPN expression is a significantly unfavourable prognostic factor for the survival of patients with stage I NSCLC. This conclusion has notable importance in terms of the biological characterization of early-stage tumours and therapeutic opportunities.

Non-small-cell lung cancer (NSCLC) accounts for approximately 75% of all cases of lung cancer, which is one of the most common tumours affecting humans in the world, and is the leading cause of cancer-related deaths for both men and women in the US. Among lung cancers, NSCLC is a subgroup of particular interest because of its heterogeneity in terms of both histopathological classification and clinical behaviour: in fact, up till now, the factors responsible for the different outcomes among patients with the same stage of disease are little known. Despite improvements in detection and in surgical and medical treatments during the past two decades, the clinical behaviour of NSCLC remains bad, with an unsatisfactory survival of even stage I patients, who develop local recurrences and distant metastases, or eventually die, in about 30–40% of the cases, within 5 years after complete surgical resection, which is currently the only potentially curative treatment for NSCLC. This observation underlines how important it is to identify novel pathological parameters in addition to disease stage and, most of all, new biological markers, in order to add further prognostic information, select high-risk patients for aggressive adjuvant treatments and set new anticancer therapies.

Osteopontin (OPN) is one of the factors that seems to be linked to cancer development, progression and metastasis in different malignancies. Osteopontin is a multifunctional protein, with a protein backbone of about 32.5 kDa and multiple sites (up to 28) of phosphorylation (Sorensen *et al*, 1995) and cleavage. Indeed, OPN takes part in a broad range of physiological events (Sodek *et al*, 2000), such as bone (Reinholt *et al*, 1990; Giachelli and Steitz, 2000) and vascular (Shijubo *et al*, 2000) remodelling and tissue repair, as well as in pathological processes, like cell-mediated immunity, dystrophic calcification, coronary stenosis, wound healing and cancer metastasis (Denhardt *et al*, 2001).

Moreover, the observation that OPN protein or gene expression levels are increased in many human tumours, including breast (Tuck *et al*, 1997; Tuck and Chambers, 2001), lung (Chambers *et al*, 1996), prostate (Thalmann *et al*, 1999), colon (Agrawal *et al*, 2002), ovarian (Kim *et al*, 2002) and gastric (Ue *et al*, 1998) cancer, confirmed the hypothesis that it plays an important role in tumorigenesis, tumour progression and metastasis (Oates *et al*, 1997; Furger *et al*, 2001). A notable consequence of the involvement of OPN in dissemination of various solid tumours is that it could be a specific target for anticancer therapy (Weber, 2001).

However, the impact of OPN on NSCLC outcome remains unclear, with discordant data in spite of the numerous studies on OPN expression conducted up till now using immunohistochemistry (IHC) and Northern blotting (Chambers *et al*, 1996; Shijubo *et al*, 1999; Zhang *et al*, 2001). In the present work, we decided to investigate OPN expression in a large series (136 cases) of stage I NSCLC, in order to clarify the role that OPN plays in these tumours' biology, and to define its usefulness as a prognostic marker in identifying subsets of patients with a high risk of recurrence and who would be suitable for adjuvant and new therapies.

## MATERIALS AND METHODS

### Patients and clinical data

A total of 136 patients with NSCLC, who consecutively underwent radical surgical resection at the Department of Cardio-Thoracic Surgery of the University of Pisa from December 1991 through December 1994, were prospectively studied. No detectable metastases in distal organs were present at the time of surgery. No patient had received chemotherapy or radiotherapy before surgery. The cohort of patients included 126 (92.6%) male and 10 (7.4%) female subjects, with a combined median age of 66 years (mean age 64.6 years; range, 45–80 years). Follow-up lasted through 30 June 2003, with a median follow-up period of 49 months for living patients (range, 2–137 months). Disease-free survival and overall survival rates were calculated as the period from surgery until the date of disease relapse or of death, respectively.

### Specimens

Neoplastic specimens were removed from the periphery of the tumour masses, since the central region of a tumour is more often subject to regressive alterations, and were formalin-fixed and paraffin-embedded for histological and immunohistochemical analysis. The pathologic features of the samples were classified according to World Health Organization histologic criteria (World Health Organization, 1982), and tumour staging was performed according to the International Union Against Cancer (UICC) and tumour-node-metastasis (TNM) classification (Mountain, 1997).

### Immunohistochemistry

Osteopontin expression was detected by IHC using an anti-OPN polyclonal antibody (R&D Systems Inc, Minneapolis, MN, USA; dilution at 1 : 40). The antibody was applied to 5 *μ*m sections taken from the most representative formalin-fixed, paraffin-embedded tumour tissue specimen obtained from each of the 136 patients with NSCLC, using the avidin–biotin–peroxidase complex method (Vectastatin Elite ABC kit; Vector Laboratories, Inc., Burlingame, CA, USA), following the manufacturer's instructions. The immunostaining was performed manually at room temperature. The sections, mounted on glass slides, were deparaffinised through serial baths in xylene, and then rehydrated in a graded series of alcohol and water. To remove any endogenous peroxidase activity and nonspecific background staining, the sections were soaked in absolute methanol containing 0.3% hydrogen peroxide for 30 min at room temperature. After being washed with TBS (Tris-Buffer Saline) for 5 min, the slides were blocked with nonimmune rabbit serum for 30 min to inhibit nonspecific binding, followed by incubation with the anti-OPN primary antibody for 60 min at room temperature. After rinsing with TBS for 5 min, the sections were subsequently incubated with biotin-conjugated goat anti-mouse IgG antibody for 30 min. Then, after being washed again with TBS for 5 min, the slides were incubated with avidin–biotin–peroxidase complex for 30 min and washed again with TBS. Finally, the sections were incubated with 0.05% 3,3′-diaminobenzidine tetrahydrochloride (Sigma, St Louis, MO, USA) and then rinsed in distilled water. All slides were lightly counterstained with Mayer's haematoxylin for 30 s, rinsed in running water, dehydrated and mounted with Canadian balsam. No antigen retrieval was performed. A section of thyroid papillary carcinoma, classic variant, previously proven to be OPN positive by Western blot, was used as positive control.

**Figure 1:**
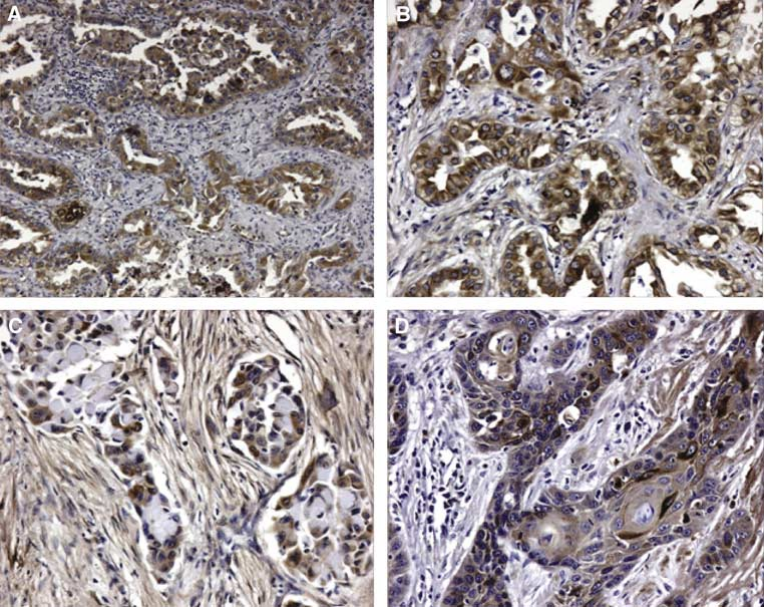
Osteopontin expression detected by immunohistochemical staining in two adenocarcinomas (**A** × 100; **B** × 200) and in two squamous-cell carcinomas (**C, D** × 200) of the lung.

The immunohistochemical expression of OPN was evaluated as the percentage of tumour cells with cytoplasmic immunoreactivity, counting at least 1000 cancer cells (100 cells in 10 HPF) for each section (Figure 1). The median value of this series (20% of positive cells) was used as the cutoff value to distinguish tumours with low (<20%) from tumours with high (⩾20%) OPN expression. Moreover, we analysed the staining intensity by distinguishing four categories: one negative (0), one with a weak staining (+), one with an intermediate staining (++) and one with a strong staining (+++).

### Statistical analysis

Statistical analysis was carried out using R 1.8.1 (R Development Core Team, 2003). Univariate analysis was performed by modelling Kaplan–Meier survival curves. Log rank test was used to evaluate the statistical significance of differences in survival distributions. Multivariate analysis was carried out using Cox proportional hazard model. The proportional hazard assumption was tested as proposed by Grambsch and Therneau (1994). Mann–Whitney and Kruskal–Wallis tests were used to evaluate the associations between the continuous-test variable-gene expression and patients' clinicopathological parameters [Mann–Whitney test for dichotomous variables (sex; age: <66 years *vs* ⩾66 years; squamous-cell carcinomas *vs* nonsquamous-cell carcinomas; relapse *vs* no relapse); Kruskal–Wallis test for nondichotomous variables (T, G)]. All tests used are described in Armitage *et al* (2002). Results were considered statistically significant if *P*<0.05.

## RESULTS

### Clinicopathological characteristics

The mean age of the 126 male patients and 10 female patients was 64.6 years (range, 45–80 years; median age, 66 years). The most common histologic type of tumour was squamous-cell carcinoma (61.8%; 84 cases), followed by adenocarcinoma (30%; 41 cases), large-cell anaplastic carcinoma (6%; eight cases) and bronchioloalveolar carcinoma (2.2%; three cases). According to the degree of differentiation, the primary tumours were histopathologically graded as well differentiated (G1, 28.7%), moderately differentiated (G2, 41.9%) and poorly differentiated (G3, 29.4%). With respect to tumour size, 54 (39.7%) cancers were classified as T1 and 82 (60.3%) were classified as T2. A total of 52 (38.23%) patients presented relapses during follow-up: 13 of them developed local recurrence, whereas 39 developed distant metastases. At the end of the follow-up, 48 patients (35.3%) were alive, while 88 (64.7%) had died.

### Association between clinicopathological characteristics and survival

In the sample of 136 stage I patients, among the clinicopathological characteristics, only tumour grade was significantly associated with disease-free survival (*P*=0.0059). Indeed, none of the other parameters showed a statistically significant correlation with outcome (Table 1).

### Association of OPN expression with clinicopathological characteristics and survival

Osteopontin expression was analysed as a dichotomous variable, using the median value of 20% as the cutoff point, and distinguishing two categories: one with a high OPN expression (⩾20%) and one with a low or null OPN expression (<20%). In total, 65 (47.8%) cases showed high OPN expression, whereas in 71 (52.2%) cases OPN expression was low. Regarding the staining intensity, 40 tumours showed a weak immunoreactivity, 36 an intermediate staining and 22 a strong staining (Table 1).

Osteopontin expression was significantly correlated with sex (*P*=0.006) and tumour grade (*P*=0.00004), and was also significantly correlated with relapse (*P*=0.02). In fact, a significantly lower OPN expression was observed in female patients (10 out of 136), in poorly differentiated (G3) tumours, and in patients who did not relapse. On the other hand, there were no significant associations of OPN expression with age (*P*=0.25) and histology (*P*=0.75) (Table 2).

Univariate analysis showed that OPN expression was significantly correlated with overall (*P*=0.034) and disease-free (*P*=0.011) survival (Table 1). In multivariate analysis, both Cox models contain only OPN expression. A high value of OPN expression is unfavourable both to survival (risk ratio 1.88 : 1) (*P*=0.037) (Table 3) and relapse (risk ratio 2.08 : 1) (*P*=0.013) (data not shown).

**Figure 2:**
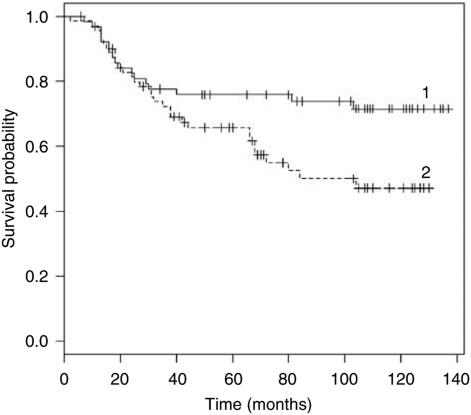
Kaplan–Meier curves for overall survival. Curve 1: Tumour stage I, low OPN expression, mean survival MS 107 months, standard error (s.e.) 6 months. Curve 2: Stage I, high OPN, MS 84 months, s.e. 6 months.

**Figure 3:**
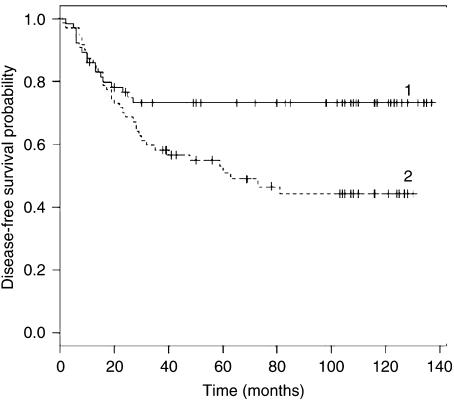
Kaplan–Meier curves for disease-free survival. Curve 1: Tumour stage I, low OPN expression, mean survival MS 104 months, standard error (s.e.) 7 months. Curve 2: Stage I, high OPN, MS 73 months, s.e. 7 months.

Figures 2 and 3 represent Kaplan–Meier curves for overall survival (Figure 2) and disease-free survival (Figure 3), respectively. Patients were divided according to high or low OPN expression. One can see that patients were significantly split by OPN expression regarding both overall (*P*=0.034) and disease-free (*P*=0.011) survival; in fact, stage I NSCLC patients with high OPN expression had shorter overall and disease-free survival than those with low OPN expression.

## DISCUSSION

The observation that OPN is involved in tumorigenesis, progression and metastatic dissemination of different types of human tumours, such as breast (Tuck *et al*, 1997; Tuck and Chambers, 2001), prostate (Thalmann *et al*, 1999), colon (Agrawal *et al*, 2002), ovarian (Kim *et al*, 2002), gastric (Ue *et al*, 1998) and lung (Chambers *et al*, 1996) cancer, has opened an interesting field of research, both for its possible prognostic significance in terms of disease-free and overall survival, and for its eventual role as a specific target in designing new anticancer therapies. Recently, an interesting study has shown an OPN immunostaining in many other tumours, such as pancreatic, renal, endometrial, oesophageal and head and neck carcinomas (Coppola *et al*, 2004).

As a consequence, in the present study, we decided to focus our interest on OPN protein expression detected by IHC in a large sample (136 cases) of NSCLCs, since these tumours still have a poor prognosis, in spite of remarkable advances in diagnosis, staging, treatment and biological characterisation. In order to establish whether OPN could be a prognostic marker for NSCLC, we analysed the correlations between OPN expression and many clinicopathological parameters, such as age and sex of patients, primary tumour size, histological type, local recurrence and, particularly, disease-free and overall survival. We decided to study in particular the subgroup of NSCLC with stage I (T1/2N0) tumours, because we think it could be very important to identify subsets of patients, who could possibly benefit from adjuvant chemoradiotherapy and, eventually, from new therapeutic trials, from among those with tumours resected in early stage.

As far as we know, our study is the largest prospective analysis of the prognostic role of OPN expression in patients with NSCLC treated with curative surgery. In our sample, OPN expression was analysed as a dichotomous variable, using the median value of 20% as the cutoff point, and distinguishing two categories: one with a high OPN expression (⩾20%) and one with a low OPN expression (<20%). In contrast to recent studies (Shijubo *et al*, 1999; Zhang *et al*, 2001; Coppola *et al*, 2004), we did not use a score, which combines the percentage of immunoreactive tumour cells with their staining intensity, because we considered this latter to be a highly subjective parameter (we evaluated staining intensity only as a descriptive parameter). According to the criteria we adopted, we found that 65 (47.8%) NSCLC had a high OPN expression, whereas in 71 (52.2%) cases OPN expression was low.

Osteopontin expression level was significantly correlated with overall and disease-free survival, both in univariate and in multivariate analyses; so, a high value of OPN expression is seen to be unfavourable both to survival (risk ratio 1.88 : 1) and relapse (risk ratio 2.08 : 1).

This result agreed with the conclusions of the study conducted on 82 NSCLCs by Schneider *et al* (2004), who showed a statistically significant association between high OPN expression and shorter survival, and with the results of one of the first works on OPN expression in 25 lung cancer patients carried out by Chambers *et al* (1996), who observed a higher OPN immunoreactivity in tumour specimens from patients who had died during follow-up.

As regards the association between OPN expression and clinicopathological parameters, such as age, sex, histological type, primary tumour size, histological grading, relapses during follow-up and status (alive *vs* dead), we observed statistically significant correlations between OPN expression and grading (*P*=0.00004), sex (*P*=0.006) and relapse (*P*=0.02).

The fact that patients with poorly differentiated (G3) tumours showed a significantly lower OPN expression than those with well-(G1) or moderately (G2) differentiated carcinomas could be explained by considering that in highly undifferentiated tumours the loss of cellular differentiation could be responsible for an increasing reduction of OPN protein expression.

The observation that OPN was expressed at lower levels in stage I female patients compared to male patients needs to be considered with caution, because in our study there was an imbalance in sample collection between men (126 cases) and women (10 cases); this imbalance did not depend on the way we selected our case history, because we chose to study a cohort of patients who had consecutively undergone surgical resection.

Interestingly, we did not describe significant differences between histological subgroups in terms of OPN expression (*P*=0.75). Our conclusion differs from those arrived at by Zhang *et al* (2001), who observed a preferential OPN expression in squamous-cell carcinomas (OPN immunoreactivity in 68.8% of squamous-cell carcinomas *vs* 20.8% of adenocarcinomas), and from those of Shijubo *et al* (1999), who described a significantly worse prognosis of stage I adenocarcinomas as compared to other groups, but matches that of the Schneider group's study (Schneider *et al*, 2004).

To sum up, in our study, a high OPN expression results as an unfavourable prognostic factor for relapse and outcome in stage I patients, and is a valid parameter by which to split this subpopulation into two groups, both for overall and for disease-free survival. This conclusion has notable importance in terms of both the biological characterisation of early-stage tumours and new therapeutic opportunities.

## Figures and Tables

**Table 1:** Univariate analysis of the associations between prognostic variables and overall survival or disease-free survival in 136 cases of stage I non-small-cell lung cancer

**Patient and tumour characteristics**	**Number of cases**	***P*-value overall survival**	***P*-value disease-free survival**
*Sex*			
Male	126		
Female	10	0.37	0.56

*Age (years)*			
⩽66	64		
>66	72	0.48	0.47

*Tumour grade (G)*			
G1	39		
G2	57		
G3	40	0.070	0.0059

*Histology*			
Squamous	84		
Nonsquamous	52	0.79	0.88

*Tumour size (T)*			
T1	54		
T2	82	0.39	0.37

*Osteopontin expression (OPN* %*)*			
Low (<20)	65		
High (⩾20)	71	0.034	0.011

*Osteopontin (OPN*+*)*			
0	38		
+	40		
++	36		
+++	22	0.11	0.090

**Table 2:** *P*-values of Mann–Whitney *t*-test and Kruskal–Wallis tests comparing OPN expression and different clinicopathological characteristics in 136 cases of stage I non-small-cell lung cancers

	**OPN %**
Sex	0.006
Age	0.25
Histology	0.75
G	0.00004
Relapse	0.02

**Table 3:** Multivariate analysis of overall survival according to Cox's model for stage I patients (final model obtained with a backward stepwise regression)

	***β***	**exp(*β*)**	**s.e. exp(*β*)**	***z***	***P*-value**
OPN % high	0.631	1.88	0.303	2.08	0.037

Likelihood ratio test=4.53 on 1 df, *P*=0.033, *n*=136. Proportional hazard test *P*=0.09. *β*=Cox regression's coefficient, exp(*β*)=risk ratio, *z*=Wald statistics, *P*=Wald's test *P*-value.
